# Supplementary material for: CdbA is a DNA-binding protein and c-di-GMP receptor important for nucleoid organization and segregation in Myxococcus xanthus
Source: Nat Commun. 2020 Apr 14;11:1791. doi: 10.1038/s41467-020-15628-8 (PMC7156744; doi:10.1038/s41467-020-15628-8)
Supplement: Supplementary file 1 — Supplementary Information [file 41467_2020_15628_MOESM1_ESM.pdf]

Supplementary Information for:

CdbA is a DNA-binding protein and c-di-GMP receptor important for nucleoid organization and segregation in *Myxococcus xanthus*

Dorota Skotnicka, Wieland Steinchen, Dobromir Szadkowski, Ian T. Cadby,
Andrew L. Lovering, Gert Bange and Lotte Søgaard-Andersen

This file includes:

Supplementary Figures 1-8

Supplementary Tables 1-4

Supplementary References

Supplementary Figures

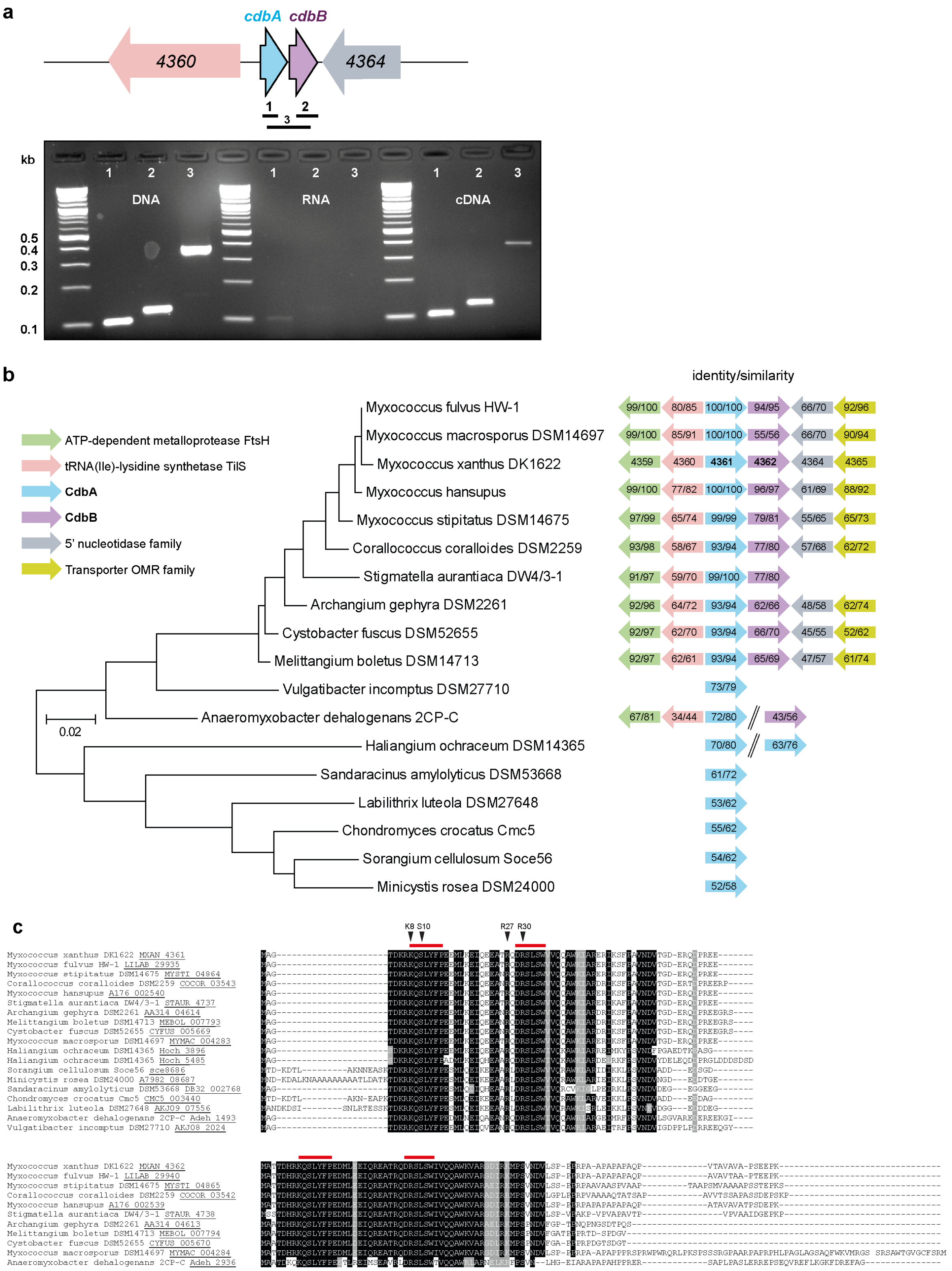

Supplementary Figure 1. CdbA and CdbB are paralogs encoded in an operon and conserved in Myxococcales

a. Operon mapping of *cdbAB* locus. Arrows indicate the direction of transcription of genes. The lines labeled 1 to 3 show fragments amplified by PCR. The PCR products were amplified using genomic DNA, cDNA and RNA as templates as indicated and separated on a 1% agarose gel. Molecular size markers in kb are shown on the left. Similar results were obtained in two independent experiments**. b.** Conservation of the *cdbAB* locus in Myxococcales. 16s rRNA sequences were aligned with ClustalW using MEGA7 and a phylogenetic tree was generated using the Maximum Likelihood method. CdbA/B homologs were identified using BLASTP analysis. Arrows indicate the direction of transcription. Numbers in the arrows indicate % identity/similarity between CdbA/B from *M. xanthus* and their homologs calculated using EMBOSS Needle software (pairwise sequence alignment). **c.** Alignment of CdbA and CdbB with their homologs from other Myxococcales with fully sequenced genomes. Sequences were aligned with ClustalW using MEGA7. Red lines indicate 100% conserved regions. Arrows above the plot indicate the position of amino acids involved in DNA- and c-di-GMP-coordination.

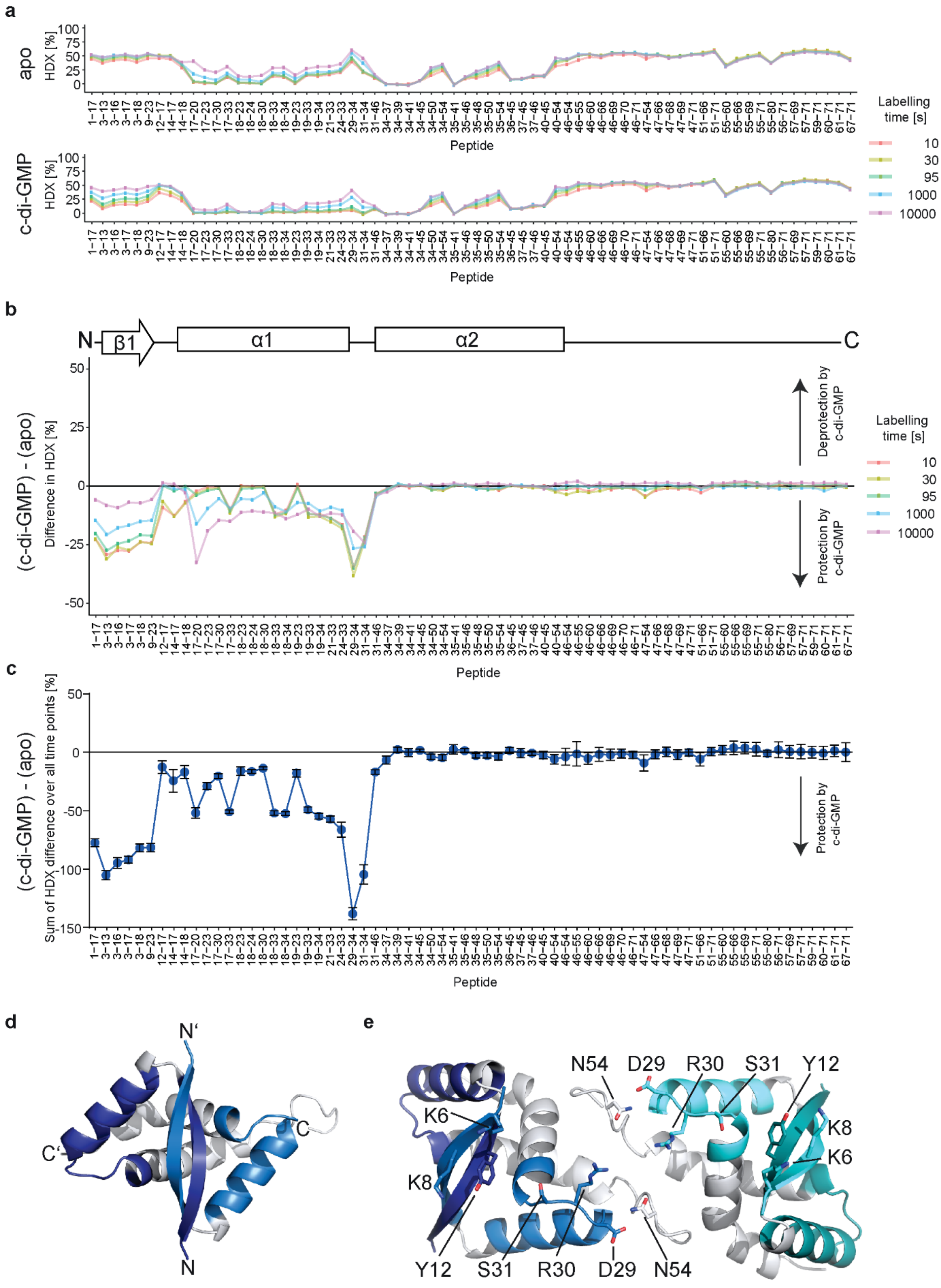
a
apo
HDX [%]
Peptide
c-di-GMP
Labelling time [s]
10
30
95
1000
10000
b
N
β1
α1
α2
C
(c-di-GMP) - (apo)
Difference in HDX [%]
Deprotection by c-di-GMP
Protection by c-di-GMP
c
Sum of HDX difference over all time points [%]
d
N'
C'
e
N54
D29
R30
S31
Y12
K6
K8

Supplementary Figure 2. HDX assay for CdbA in the presence and absence of c-di-GMP. **a.** Relative HDX profiles of CdbA in the apo-state (top) and c-di-GMP-bound state (bottom). **b.** Difference between the relative HDX of c-di-GMP-bound and apo-CdbA. The secondary structure of CdbA is depicted above the plot. **c.** The differences in HDX between c-di-GMP-bound and apo-CdbA at each time point (i.e. 10, 30, 95, 1000 and 10000 s) were summed for each peptide. The graph depicts the summed means of differences ± SD (n=3 separate reactions) for each peptide. **d.** Peptides with reduced (>0.5 Da difference) HDX in the c-di-GMP-bound state of CdbA illustrated on the CdbA dimer and colored in dark and light blue for the respective monomer. **e.** Peptides with reduced (>0.5 Da difference) HDX in the c-di-GMP-bound state of CdbA illustrated on the CdbA tetramer. Amino acids lining the polar pocket established at the dimer/dimer interface are shown as sticks. Plots of hydrogen/deuterium exchange profiles were generated with MEMHDX[1].

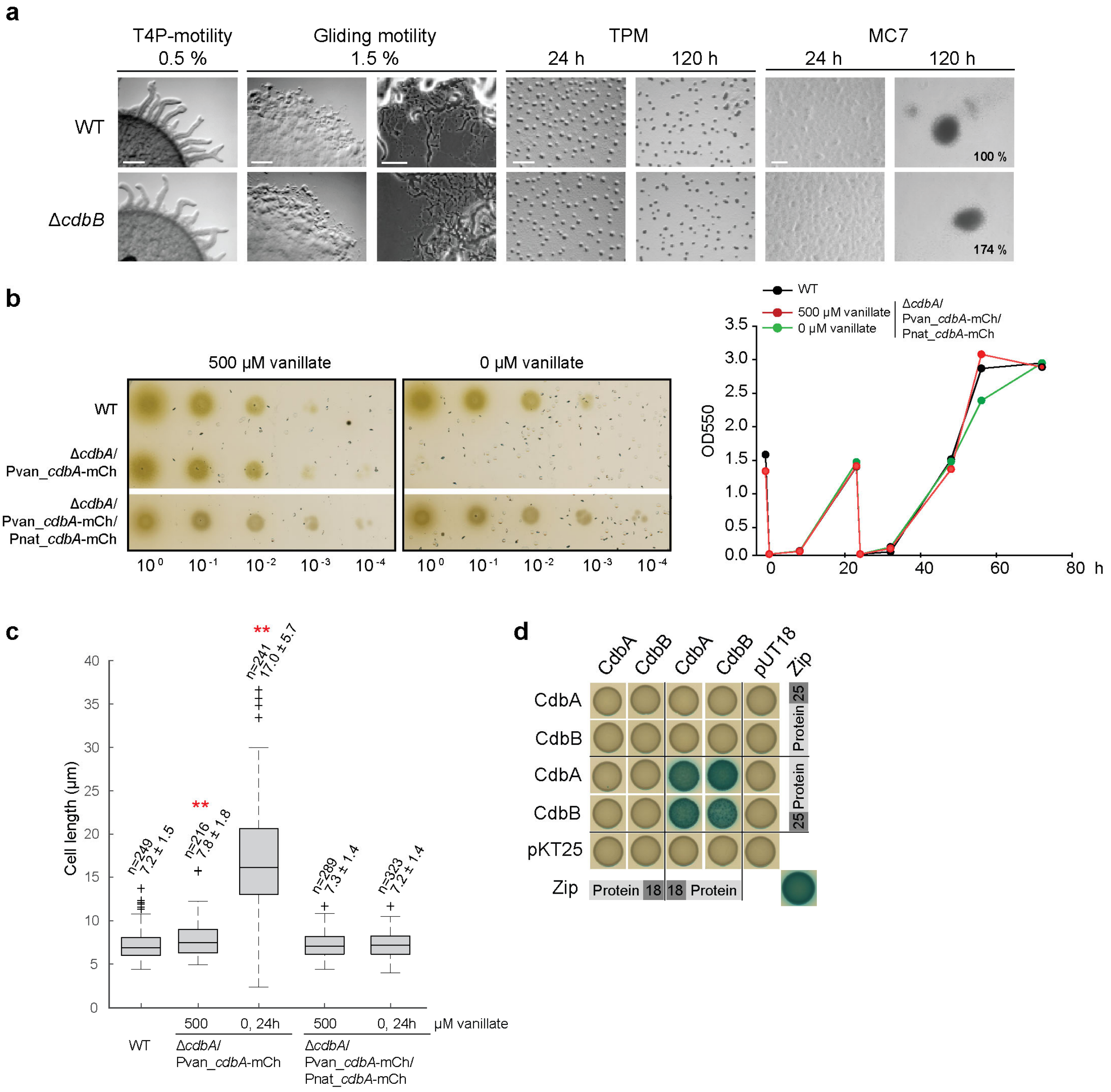

Supplementary Figure 3. CdbB is not important for motility, growth and development; CdbA-mCherry is active and CdbA and CdbB interact in BACTH.

a. Lack of CdbB causes no defects in motility and development. T4P-dependent motility and gliding motility were analyzed on 0.5% and 1.5% agar, respectively. Fruiting body formation and sporulation were analyzed under two different starvation conditions: TPM agar and MC7 submerged culture. Numbers for submerged culture indicate heat- and sonication resistant spores formed after 120h of starvation as a percentage of WT (100%). Experiment was repeated twice with similar result. Scale bars, 1 mm (0.5% agar), 1 mm (1.5% agar, left), 50 µm

(1.5% agar, right), 1 mm (TPM agar), 100 µm (MC7). **b.** CdbA-mCherry is active. Growth of indicated strains on solid surface (left) and in suspension (right) in the presence and absence of vanillate driving synthesis of CdbA. Results for WT, Δ*cdbA*/Pvan_*cbdA*-mCh and Δ*cdbA*/Pvan_*cbdA*-mCh/Pnat_*cbdA*-mCh are the same as in Fig. 6e and all the strains were spotted on the same plate. Similar results were obtained in two independent experiments. Source data are provided as a Source Data file. **c.** Cell length distributions of cells of indicated genotypes grown in the presence or absence of vanillate. In the boxplots, boxes enclose the 25th and 75th percentile with the black line representing the mean, whiskers indicate the 10th and 90th percentile, and "+" indicates outliers. Numbers above each box indicate number of cells used for quantification (n) and mean ± SD. ** $p<0.001$ in two-sided Student's t-test, in comparison to WT. Exact p-values and source data are provided in the Source data file. **d.** Bacterial Two-hybrid analysis of interactions between CdbA and CdbB. The indicated proteins were fused to the N or C terminus of the T25 and T18 fragments of CyaA. Zip indicates the leucine zipper from GCN4 fused to T25 and T18, used as a positive control. Experiment was repeated twice with similar result. Source data are provided as a Source Data file.

a

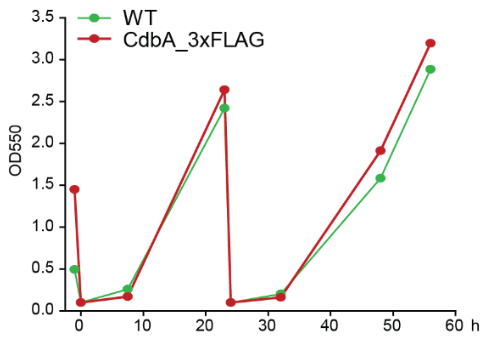

b

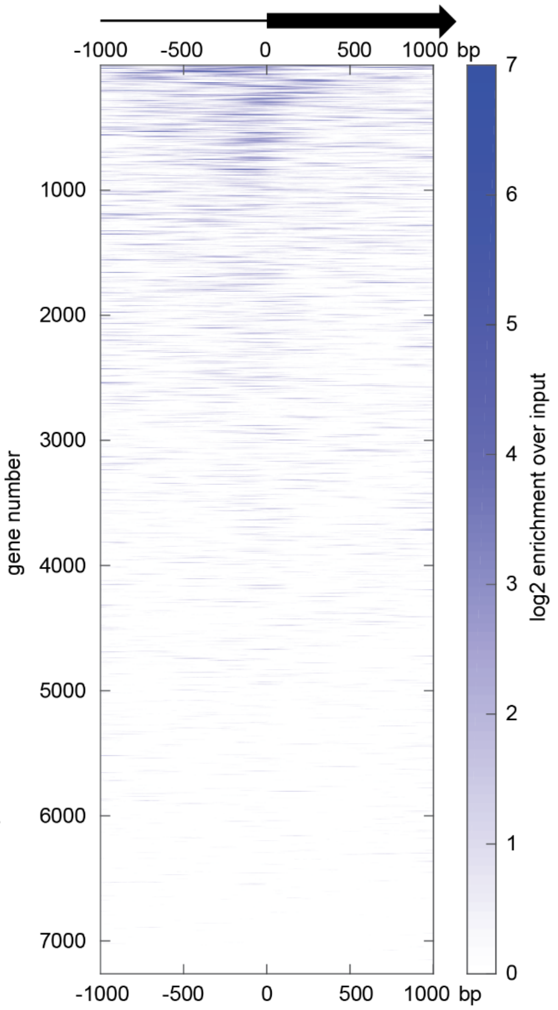

c

Motif p-value	Peak enrichment (log2)	Number of peaks	Motifs found genome wide	Peaks with at least 1 motif
<0.00077	≥2	569	8917	296
<0.00044	≥2	569	2906	220
<0.000088	≥2	569	799	136

d

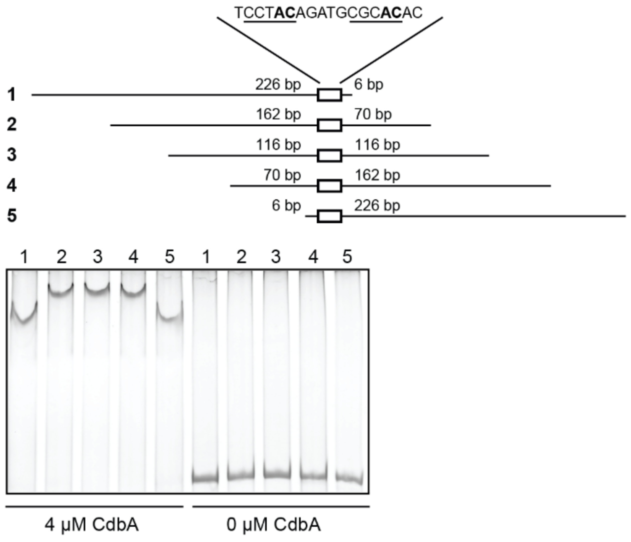

e

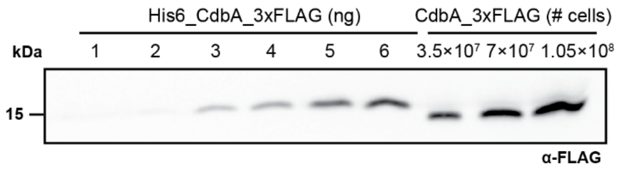

Supplementary Figure 4. Supplementary information for ChIP-seq experiment

a. C-terminal 3×FLAG tag does not affect CdbA function. Growth of WT and CdbA-3×FLAG strains in liquid culture. Experiment was repeated twice with the similar results. Source data are provided as a Source Data file. **b.** Enrichment of top CdbA ChIP-seq peaks in promoter regions.

Each row in the heatmap represents 1000 bp starting from each *M. xanthus* ORF (oriented as shown in the gene cartoon, such that the start codon occupies the same position in each row), as well as the 1000 bp upstream of the start site of each ORF. Color reflects the degree of CdbA ChIP-seq signal enrichment over input in $\log_2$ (bluer color = greater enrichment). Rows were sorted by $\log_2$ of CdbA ChIP-seq signal enrichment over input from highest (top) to lowest (bottom). **c.** Correlation between ChIP-seq peaks and MEME-ChIP-based direct repeat identified in *M. xanthus* genome *in silico*. **d.** Circular permutation analysis of DNA bending by CdbA. The left panel shows DNA fragments generated by PCR where CdbA binding motif (direct repeat) is located at the different positions. The right panel shows the results of the gel shift experiment with/without CdbA and circularly permutated DNA fragments (1 to 5). Fragments with the binding site closer to the center migrate slower than the fragments which have the site closer to the ends. Experiment was repeated twice with similar results. Source data are provided as a Source Data file. **e.** Quantification of CdbA molecules per cell. Different amounts (1-6 ng) of purified His_6-CdbA-3×FLAG were loaded in parallel with cell extracts from a known number of WT cells (3 different dilutions) and probed with α-FLAG antibodies. Number of molecules per cell was calculated as an average of two biological replicates from the intensity of the lysates bands in comparison to a standard curve prepared from the dilution series of known purified protein amounts from the same immunoblot. Source data are provided as a Source Data file.

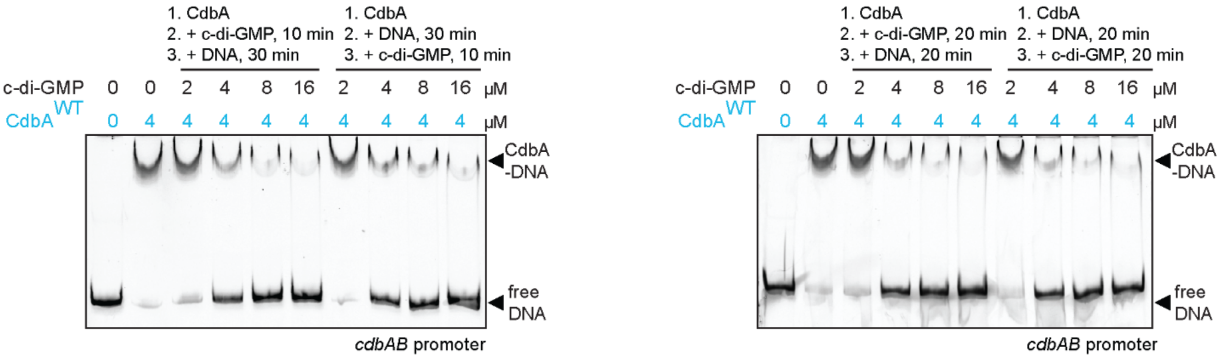

Supplementary Figure 5. DNA binding by CdbA is inhibited by c-di-GMP independently of the order of addition of c-di-GMP and DNA

EMSA experiment with CdbA in the presence of c-di-GMP. The DNA fragment used covers the *cdbAB* promoter (see also Fig. 7a). Left panel, 4 µM CdbA was incubated with the indicated concentrations of c-di-GMP for 10 min and then with DNA for 30 min, or with DNA for 30 min and then with c-di-GMP for 10 min. Right panel, 4 µM CdbA was incubated with the indicated concentrations of c-di-GMP for 20 min and then with DNA for 20 min, or with DNA for 20 min and then with c-di-GMP for 20 min. Similar results were obtained in two independent experiments. Source data are provided as a Source Data file.

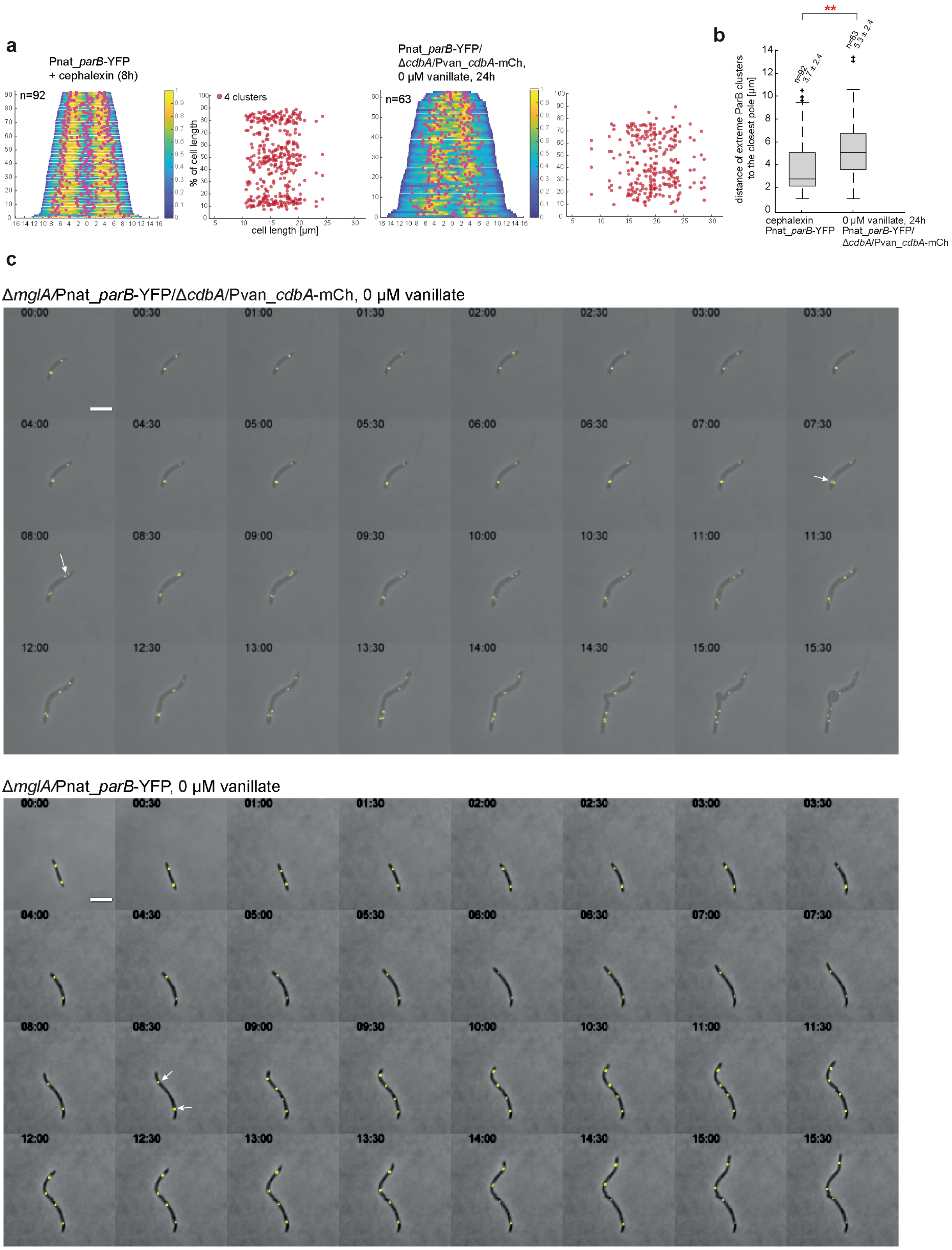
a
Pnat_parB-YFP
+ cephalexin (8h)
n=92
4 clusters
% of cell length
cell length [µm]
Pnat_parB-YFP/
ΔcdbA/Pvan_cdbA-mCh,
0 µM vanillate, 24h
n=63
b
**
n=92
3.7 ± 2.4
n=63
5.3 ± 2.4
distance of extreme ParB clusters
to the closest pole [µm]
cephalexin
Pnat_parB-YFP
0 µM vanillate, 24h
Pnat_parB-YFP/
ΔcdbA/Pvan_cdbA-mCh
c
ΔmglA/Pnat_parB-YFP/ΔcdbA/Pvan_cdbA-mCh, 0 µM vanillate
ΔmglA/Pnat_parB-YFP, 0 µM vanillate

Supplementary Figure 6. Lack of CdbA affects segregation of ParB-YFP clusters.

a. Same analysis as in Fig. 7a but only including cells with four ParB-YFP clusters: demographs showing overlay of DAPI staining and ParB-YFP localization and scatter plot showing the position of ParB-YFP clusters along the long cell axes as a function of cell length. n is indicated for each strain. **b.** Same analysis as in Fig. 7c but only including cells with four ParB-YFP clusters: box plot showing distance of extreme ParB clusters to the closest pole as a function of cell length. In the boxplots, boxes enclose the 25th and 75th percentile with the black line representing the mean, whiskers indicate the 10th and 90th percentile, and "+" indicates outliers. Numbers above each box indicate number of cells used for quantification (n) and mean ± SD. ** $p<0.001$ in two-sided Student's t-test. Exact p-values and source data are provided in the Source data file. **c.** Time-lapse images of ParB-YFP localization and segregation. For time-lapse microscopy, non-motile strains were created by deletion of *mglA* resulting in a complete loss of motility[2]. Cells of Δ*mglA/*Pnat_*parB*-YFP/Δ*cdbA*/Pvan_*cbdA*-mCh grown in the presence of 500 μM vanillate were washed, spotted on agar pads without vanillate and imaged every 0.5 h for 15.5 h. As a control, cells of Δ*mglA/*Pnat_*parB*-YFP were used under the same conditions, without vanillate. White arrows indicate duplication event of ParB-YFP followed by rapid segregation in the Δ*mglA/*Pnat_*parB*-YFP strain and slow segregation in the Δ*mglA/*Pnat_*parB*-YFP/Δ*cdbA*/Pvan_*cbdA*-mCh strain. Representative cells are shown. Scale bars, 5 μm. Similar results were obtained in two independent experiments.

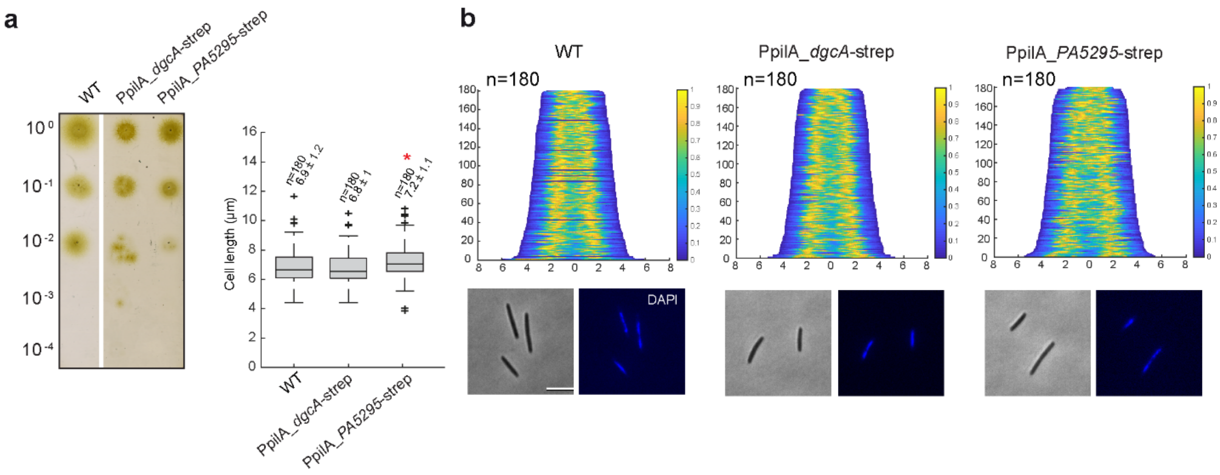

Supplementary Figure 7. Manipulating c-di-GMP level does not visibly affect chromosome organization.

a. Left panel: growth of indicated strains on solid surface. All the strains were spotted on the same plate. Experiment was repeated twice and representative image is shown. Right panel: box plot showing cell length distributions of cells of indicated genotypes. In the boxplots, boxes enclose the 25th and 75th percentile with the black line representing the mean, whiskers indicate the 10th and 90th percentile, and "+" indicates outliers. Numbers above each box indicate number of cells used for quantification (n) and mean ± standard deviation. * p<0.05 in a two-sided Student's t-test, in comparison to WT. Exact p-values and source data are provided in the Source data file. **b.** Upper panel: demographs showing DAPI staining of cells of indicated genotypes. n=180 for each strain. Lower panel: images of representative cells used for the analysis. Scale bar, 5 µm.

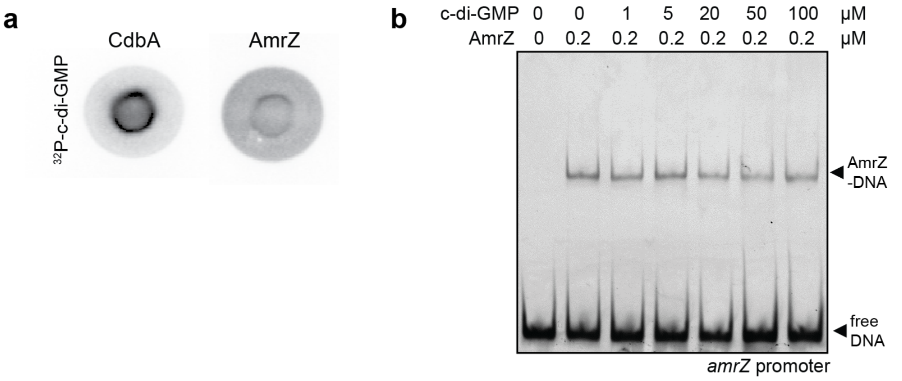

Supplementary Figure 8. Ribbon-helix-helix protein AmrZ does not bind c-di-GMP and c-di-GMP does not affect AmrZ-DNA binding

a. Purified AmrZ-His_6 does not bind ^{32}P-c-di-GMP in DRaCALA. Purified CdbA-His_6 was used as a positive control. Experiment was repeated twice with the same result. Source data are provided as a Source Data file. **b.** c-di-GMP has no effect on AmrZ binding to its promoter in EMSA. Concentration of AmrZ and c-di-GMP is indicated for each lane. Experiment was repeated twice with the same result. Source data are provided as a Source Data file.

Supplementary Table 1.

Data collection and refinement statistics for CdbA structure

	Pt Derivative	Form 1	Form 2
**Data collection**			
Space group	$I4_122$	$I4_122$	$I4_122$
Cell dimensions *a*, *b*, *c* (Å)	61.69, 61.69, 92.52	61.47, 61.47, 94.91	134.69, 134.69, 53.13
Resolution (Å)	3.2	2.24	2.33
Wavelength	1.0721	1	1
R_{merge}	0.09 (0.73)*	0.05 (1.34)	0.09 (1.60)
$I / \sigma I$	53.4 (9.1)	19.6 (1.7)	17.5 (1.6)
CC(1/2)	100 (100)	100 (60.0)	99.9 (64.7)
Completeness (%)	99.3 (100)	100 (100)	99.4 (95.6)
Redundancy	67.2 (49.3)	11.1 (5.6)	23.6 (19.2)
**Refinement**			
Resolution (Å)		2.24	2.33
R_{work} / R_{free}		23.2/27.7	21.6/24.4
R.m.s. deviations			
Bond lengths (Å)		0.006	0.006
Bond angles (°)		1.37	1.55

*Values in parentheses are for highest-resolution shell.

Supplementary Table 2.

Strains used in this work

Strain	Genotype	Reference
M. xanthus		
DK1622	Wild-type	[3]
SA3535	*attB::*pTP110 (P*pilA*-PA5295-strepII)	[4]
SA3543	*attB::*pTP114 (P*pilA-dgcA*-strepII)	[4]
SA4749	Δ*mglA*; *attB::*pAH07 (Pnat *parB*-YFP)	[5]
SA5645	Δ*cdbB*	This work
SA5685	*attB::*pDJS125 (Pnat *cdbA*-mCherry)	This work
SA5690	Δ*cdbA*; *mxan*18-19::pDJS148 (Pvan *cdbA*-Cherry)	This work
SA5691	SA5690; *attB::*pDJS151 (Pnat *parB*-YFP)	This work
SA5693	*attB::*pDJS151 (Pnat *parB*-YFP)	This work
SA5697	SA5690; *attB::*pDJS125 (Pnat *cdbA*-mCherry)	This work
SA8802	SA5690; *attB::*pDJS154 (Pnat *cdbA*$^{K8A/S10A}$-mCherry)	This work
SA8805	SA5690; *attB::*pDJS155 (Pnat *cdbA*$^{R27A/R30A}$-mCherry)	This work
SA8810	Δ*cdbA*Δ*cdbB*; *mxan*18-19::pDJS148 (P_{van} *cdbA*-Cherry)	This work
SA8813	*cdbA*_3xFLAG; native site	This work
SA8814	SA5690; Δ*mglA; attB::*pDJS151 (Pnat *parB*-YFP)	This work
SA8834	*parB*_3xFLAG; native site	This work
E. coli		
BL21(DE3)	F- *ompT hsdSB (rB- mB-) gal dcm* (DE3)	Invitrogen
Mach1	F-Φ80*lac*ZΔM*15*Δ*lac*X74*hsd*R(rK–, mK+)Δ*recA*1398 *end*A1 *ton*A	Invitrogen
Rosetta2(DE3)	F- *ompT hsd*SB (rB- mB-) *gal dcm* (DE3) pRARE2 (CamR)	Novagene
BTH101	F- *cya*-99 *araD*139 *galE*15 *galK*16 *rpsL1* (Str^r) *hsdR*2 *mcrA*1 *mcrB*1	Euromedex

Supplementary Table 3.

Plasmids used in this work

Plasmid	Description	Reference
pET24b(+)	Expression vector, Kan^R	Novagen
pET28a(+)	Expression vector, Kan^R	Novagen
pRSFDuet-1	Dual expression vector, Kan^R	Novagen
pBJ114	$Kan^{R,}$ *galK*	[6]
pSW105	P*pilA,* Kan^R	[7]
pSWU19	Kan^R	[8]
pMR3691	*vanR*-$P_{van,}$ Tet^R	[9]
pKT25	Two-hybrid plasmid, *cyaAT25* C-terminal fusion, Km^R	Euromedex
pUT18	Two-hybrid plasmid, *cyaAT18* N-terminal fusion, Amp^R	Euromedex
pKNT25	Two-hybrid plasmid, *cyaAT25* N-terminal fusion, Km^R	Euromedex
pUT18C	Two-hybrid plasmid, *cyaAT18* C-terminal fusion, Amp^R	Euromedex
pKT25-zip	Two-hybrid control plasmid Km^r	Euromedex
pUT18C-zip	Two-hybrid control plasmid Amp^r	Euromedex
pDJS31	pET24b(+); *dgcA*_His_6, Kan^R	[4]
pAH07	pSWU30, Pnat *parB*-YFP, Tet^R	[10]
pSL16	pBJ114; in-frame deletion construct for *mglA,* Kan^R	[2]
pNG62	pSWU19; MCS-linker-mCherry, Kan^R	This work
pDJS83	pET24b(+); *cdbB*_His_6, Kan^R	This work
pDJS85	pBJ114; in-frame deletion construct for *cdbA-B,* Kan^R	This work
pDJS86	pET24b(+); *cdbA*_His_6, Kan^R	This work
pDJS97	pBJ114; in-frame deletion construct for *cdbB,* Kan^R	This work
pDJS99	pBJ114; in-frame deletion construct for *cdbA,* Kan^R	This work
pDJS105	pET24b(+); *amrZ*_His_6, Kan^R	This work
pDJS107	pET24b(+); *cdbA*$^{K8A/S10A}$_His_6, Kan^R	This work
pDJS125	pNG62; Pnat *cdbA*-mCherry, Kan^R	This work
pDJS127	pET24b(+); *cdbA*$^{R27A/R30A}$_His_6, Kan^R	This work
pDJS129	pSW105; Pnat *cdbA*$^{K8A/S10A}$, Kan^R	This work
pDJS130	pSW105; Pnat *cdbA*$^{R27A/R30A}$, Kan^R	This work
pDJS132	pKT25, *cyaT25 – cdbA,* Km^R	This work
pDJS133	pKNT25, *cdbA - cyaT25,* Km^R	This work
pDJS134	pUT18, *cdbA - cyaT18,* Amp^R	This work
pDJS135	pUT18C, *cyaT18 – cdbA,* Amp^R	This work
pDJS136	pKT25, *cyaT25 – cdbB,* Km^R	This work
pDJS137	pKNT25, *cdbB - cyaT25,* Km^R	This work
pDJS138	pUT18, *cdbB - cyaT18,* Amp^R	This work

pDJS139	pUT18C, *cyaT18 – cdbB,* Amp^R	This work
pDJS143	pRSFDuet-1; *cdbA*-mCherry, Kan^R	This work
pDJS148	pMR3691; *cdbA*-mCherry, Tet^R	This work
pDJS151	pSWU19, Pnat *parB*-YFP, Kan^R	This work
pDJS154	pNG62; Pnat *cdbA*$^{K8A/S10A}$-mCherry, Kan^R	This work
pDJS155	pNG62; Pnat *cdbA*$^{R27A/R30A}$-mCherry, Kan^R	This work
pDJS170	pBJ114; *cdbA*_3xFLAG; native site, Kan^R	This work
pDJS177	pET28a(+); His_6_*cdbA*_3xFLAG, Kan^R	This work
pDJS179	pBJ114; *parB*_3xFLAG; native site, Kan^R	This work

Supplementary Table 4.

Primers used in this work

Primer name	Sequence (5'-3')
**Cloning primers**	
4362 F	ATCGCATATGATGGCTACGACGGACCATCGT
4362 -stop R	ATCGAAGCTTCTTGGGCTCTTCGGAGGGCGC
4361-2_A	ATCGGGTACCTTCCCATCAGGAGGGCCGTGG
4361-2_B (5aa)	GGGCTCTTCGTCGGTGCCTGCCATACT
4361-2_C (5aa)	GGCACCGACGAAGAGCCCAAGTAGTTC
4361-2_D	ATCGTCTAGAGCGCCAGGAAGGTCTGTGCGT
4361 Fw	ATCGCATATGATGGCAGGCACCGACAAGCGC
4361 -stop Rev	ATCGAAGCTTCTCCTCCCGAGGGTCCTGGCG
4362_A	ATCGGGTACCGAGGCACGTGCGGTTGAAGGC
4362_B 5aa	GGGCTCTTCGTCCGTCGTAGCCATCCG
4362_C 5aa	ACGACGGACGAAGAGCCCAAGTAGTTC
4361_B 5aa	CTCCCGAGGGTCGGTGCCTGCCATACT
4361_C 5aa	GGCACCGACCCTCGGGAGGAGTAACGA
4361_D 5aa	ATCGTCTAGATGCGCGAACTGACGGTGGGTG
4361 AQA Fw	ATCGCATATGATGGCAGGCACCGACAAGCGC**GCG**CAG**GCG**CTGTA
4361 R27,30A F	GAGGCGACC**GCG**CAGGAC**GCG**TCCCTTTCC
4361 R27,30A R	GGAAAGGGA**CGC**GTCCTG**CGC**GGTCGCCTC
Pnat 4361 F KpnI	ATCGGGTACCCGGACCGCGGTGGAATGGAG
4361 -st R BamHI	ATCGGGATCCCTCCTCCCGAGGGTCCTGGC
4361 AQA +	CGACAAGCGC**GCG**CAG**GCG**CTGTACTTCCC
4361 AQA -	GGGAAGTACAG**CGC**CTG**CGC**GCGCTTGTCG
DSZ 24	ATCGGGTACCCTACTTGTACAGCTCGTCCATGC
4361-2 Fw	ATCGCATATGGCAGGCACCGACAAGCGCAAG
GFP rv_STOP_EcoRI	ATCGGAATTCTTACTTGTACAGCTCGTCCAT
DA-325	ACACCGCATATGGAGGCGTGTC
KA439	GCGTCTAGATCACTTGTACAGCTCGTCCATGCC
4361 3xFLAG fw	TACAAGGACCACGACATCGACTACAAGGACGACGACGACAAGTGACGACGGATGGCTACGACGGA
4361 3xFLAG rev	GTCGATGTCGTGGTCCTTGTAGTCGCCGTCGTGGTCCTTGTAGTCCTCCTCCCGAGGGTCCTGGC
4361 no start NdeI	ATCGCATATGGCAGGCACCGACAAGCGCAA
4361 3xFLAG stop BamHI	ATCGGGATCCTCACTTGTCGTCGTCGTCCT
ParB 3xFLAG A	ATCGGAATTCAAGCTCCCCATCGAGTCCAT
ParB 3xFLAG B	GTCGATGTCGTGGTCCTTGTAGTCGCCGTCGTGGTCCTTGTAGTCCTCCTTCCTGAGAAGCTTCA

ParB 3xFLAG C	TACAAGGACCACGACATCGACTACAAGGACGACGACGACAAGTGA GACGTGGCGCTCCTTGGCGG
ParB 3xFLAG D	ATCGTCTAGACTCGCGGACGCGGTCGACCA
amrZ F	ATCGCATATGATGCGCCCACTGAAACAGGC
amrZ -stop R	ATCGAAGCTTGGCCTGGGCCAGCTCCGCAT
BTH 4361 XbaI fw pKT25/pUT18C	ATCGGTCTAGAGATGGCAGGCACCGACAAGCGC
BTH 4361 KpnI rev stop pKT25/pUT18C	ATCGGGGTACCTTACTCCTCCCGAGGGTCCTG
BTH 4361 XbaI fw pKNT25/pUT18	ATCGGTCTAGAATGGCAGGCACCGACAAGCGC
BTH 4361 KpnI rev pKNT25/pUT18	ATCGGGGTACCGCCTCCTCCCGAGGGTCCTGGCG
BTH 4362 XbaI fw pKT25/pUT18C	ATCGGTCTAGAGATGGCTACGACGGACCATCGT
BTH 4362 KpnI rev stop pKT25/pUT18C	ATCGGGGTACCCTACTTGGGCTCTTCGGAGGG
BTH 4362 XbaI fw pKNT25/pUT18	ATCGGTCTAGAATGGCTACGACGGACCATCGT
BTH 4362 KpnI rev pKNT25/pUT18	ATCGGGGTACCGCCTTGGGCTCTTCGGAGGGCGC
**EMSA primers**	
4361 up250 HEX F	[HEX] CCACGGGCCCGCCTGGAGAT
4361 up R	ACTTGGGTCTCCGGGGCGGA
HEX 7438 F	[HEX] GGTAAGGGTGACGATGCC
7438 qPCR rev	ACGTACACCACCGAGTCCTT
EMSA amrZ HEX fw	[HEX] AACGACCGGTGGTCAGAAGG
EMSA amrZ rev	GTCAATTGTGCGTTGCGTGC
EMSA 4058 HEX fw	[HEX] GACACACCTTCTCCGAGCGG
EMSA 4058 rev	GTCGAGCTTCTCGCGGTTGA
EMSA 4328 HEX fw	[HEX] AGGAAGAGGGCGGCGGCGAG
EMSA 4328 rev	CTTGTCGAAACGGAGGTGGG
EMSA 4058 motif mut +	AGAATGT**AC**GCGCATCT**AC**AGGAGGTCG
EMSA 4058 motif mut -	CGACCTCCT**GT**AGATGCGC**GT**ACATTCT
EMSA 4058 motif mut half+	AGAATGT**GT**GCGCATCT**AC**AGGAGGTCG
EMSA 4058 motif mut half -	CGACCTCCT**GT**AGATGCGC**AC**ACATTCT
**Circular permutation assay primers**	
bending_1_fw	CTGGGCCTGCTGATCAGGCCGTTT
bending_1_rev	GCGACCTCCTACAGATGCGCACAC
bending_2_fw	GACACACCTTCTCCGAGCGGCTTG
bending_2_rev	GTCGAGCTTCTCGCGGTTGAGCCG

bending_3_fw	TGCGCTCGGGTTCGGCGCGGGCCT
bending_3_rev	ACTCGCTCTGACGGATGCGCGTGG
bending_4_fw	CACTGGGGTTCAGTGGATCTTCCC
bending_4_rev	GCTCTTCGTACTTCGCGAGCAGAT
bending_5_fw	GAGAATGTGTGCGCATCTGTAGGA
bending_5_rev	GAGCCACCCTTCCACATCGGGTAA
**RT-PCR primers**	
4362 qPCR forw	CCGAGGACATGCTGGAGGAG
4362 qPCR rev	CGTTGACCGACGGCATCTTC
4328 qPCR forw	CCGTTTCGACAAGGTCTTCA
4328 qPCR rev	GGACCTCCACGAACATGC
4058 qPCR forw	GACGCCCTCAAGGACCTG
4058 qPCR rev	TTCGTACTTCGCGAGCAGAT
5589 qPCR forw	CTCGGTGATGAGCCAGGT
5589 qPCR rev	CTGCAGCCCATCCTTGATGA
3850 qPCR forw	CTACGAGGAGATCGGCAAGA
3850 qPCR rev	TTGCCCTCTTCGATGAGCTT
4361 qPCR forw	CACCGACAAGCGCAAGCAG
4361 qPCR rev	GCACGACCCAGGAAAGGGA
0698 qPCR forw	CGTCATCGACAACGTGAAGA
0698 qPCR rev	GCCTGTTTGTAGTTGCCCTT
5498 qPCR forw	GACGGTCGAGTTCGAGATTG
5498 qPCR rev	GTGCAGGTCCAGTTCGATG
6587 qPCR forw	GAGCGCCTTCGTGAACAA
6587 qPCR rev	CTCATTCCCGCCGTGTCG
6483 qPCR forw	GTCATCCTCTCGGTGCTCTC
6483 qPCR rev	CCCGTCTTGCCTTCAATGG

* Restriction sites are underlined and mutations introduced by site-directed mutagenesis are in bold.

Supplementary References

1 Hourdel, V. *et al.* MEMHDX: an interactive tool to expedite the statistical validation and visualization of large HDX-MS datasets. *Bioinformatics* **32**, 3413-3419, doi:10.1093/bioinformatics/btw420 (2016).

2 Miertzschke, M. *et al.* Structural analysis of the Ras-like G protein MglA and its cognate GAP MglB and implications for bacterial polarity. *EMBO J* **30**, 4185-4197, doi:10.1038/emboj.2011.291 (2011).

3 Kaiser, D. Social gliding is correlated with the presence of pili in *Myxococcus xanthus*. *Proc Natl Acad Sci USA* **76**, 5952-5956, doi:10.1073/pnas.76.11.5952 (1979).

4 Skotnicka, D. *et al.* c-di-GMP regulates type IV pili-dependent-motility in *Myxococcus xanthus*. *J Bacteriol*, doi:10.1128/JB.00281-15 (2015).

5 Schumacher, D. & Søgaard-Andersen, L. Fluorescence live-cell imaging of the complete vegetative cell cycle of the slow-growing social bacterium *Myxococcus xanthus*. *J Vis Exp*, doi:10.3791/57860 (2018).

6 Julien, B., Kaiser, A. D. & Garza, A. Spatial control of cell differentiation in *Myxococcus xanthus*. *Proc Natl Acad Sci USA* **97**, 9098-9103, doi:10.1073/pnas.97.16.9098 (2000).

7 Jakovljevic, V., Leonardy, S., Hoppert, M. & Søgaard-Andersen, L. PilB and PilT are ATPases acting antagonistically in type IV pilus function in *Myxococcus xanthus*. *J Bacteriol* **190**, 2411-2421, doi:10.1128/JB.01793-07 (2008).

8 Wu, S. S. & Kaiser, D. Genetic and functional evidence that type-IV pili are required for social gliding motility in *Myxococcus xanthus*. *Mol Microbiol* **18**, 547-558, doi:10.1111/j.1365-2958.1995.mmi_18030547.x (1995).

9 Iniesta, A. A., Garcia-Heras, F., Abellon-Ruiz, J., Gallego-Garcia, A. & Elias-Arnanz, M. Two systems for conditional gene expression in *Myxococcus xanthus* inducible by isopropyl-beta-D-thiogalactopyranoside or vanillate. *J Bacteriol* **194**, 5875-5885, doi:10.1128/JB.01110-12 (2012).

10 Treuner-Lange, A. *et al.* PomZ, a ParA-like protein, regulates Z-ring formation and cell division in *Myxococcus xanthus*. *Mol Microbiol* **87**, 235-253, doi:10.1111/mmi.12094 (2013).
