# Supplementary material for: CdbA is a DNA-binding protein and c-di-GMP receptor important for nucleoid organization and segregation in Myxococcus xanthus
Source: Nat Commun. 2020 Apr 14;11:1791. doi: 10.1038/s41467-020-15628-8 (PMC7156744; doi:10.1038/s41467-020-15628-8)
Supplement: Supplementary file 3 — Description of Additional Supplementary Files [file 41467_2020_15628_MOESM3_ESM.pdf]

Description of Additional Supplementary Files

File Name: Supplementary Data 1
Description: Overview of data obtained by hydrogen-deuterium exchange mass spectrometry (HDX-MS).

File Name: Supplementary Data 2
Description: ChIP-seq peaks. The list of ChIP-seq peaks from the CdbA_3xFLAG strain from one replicate showing peak rank, peak coordinates, coordinates of peak summit, peak length, peak shape score, p-value and enrichment over input for all the peaks with enrichment over input > 4-fold, sorted based on peak shape score. Absolute positions indicates if peak summit was found in the coding or intergenic region. Additionally, for each peak distance of peak summit to the closest ORF start codon and position of peak summit relatively to the closest ORF start codon has been calculated and the gene name with its predicted function in old and new annotation has been assigned. For each peak it was indicated by “YES” or “NO” if a putative CdbA binding motif could be found in a range of peak summit +/-50 bp using a Hidden Markov Model.
