# Supplementary material for: CdbA is a DNA-binding protein and c-di-GMP receptor important for nucleoid organization and segregation in Myxococcus xanthus
Source: Nat Commun. 2020 Apr 14;11:1791. doi: 10.1038/s41467-020-15628-8 (PMC7156744; doi:10.1038/s41467-020-15628-8)
Supplement: Supplementary file 6 — Reporting Summary [file 41467_2020_15628_MOESM6_ESM.pdf]

nature research

Corresponding author(s): Lotte Søgaard-Andersen

Last updated by author(s): Mar 13, 2020

Reporting Summary

Nature Research wishes to improve the reproducibility of the work that we publish. This form provides structure for consistency and transparency in reporting. For further information on Nature Research policies, see Authors & Referees and the Editorial Policy Checklist .

Statistics

For all statistical analyses, confirm that the following items are present in the figure legend, table legend, main text, or Methods section.

n/a	Confirmed	
☐	☒	The exact sample size (*n*) for each experimental group/condition, given as a discrete number and unit of measurement
☐	☒	A statement on whether measurements were taken from distinct samples or whether the same sample was measured repeatedly
☐	☒	The statistical test(s) used AND whether they are one- or two-sided<br>*Only common tests should be described solely by name; describe more complex techniques in the Methods section.*
☒	☐	A description of all covariates tested
☒	☐	A description of any assumptions or corrections, such as tests of normality and adjustment for multiple comparisons
☐	☒	A full description of the statistical parameters including central tendency (e.g. means) or other basic estimates (e.g. regression coefficient) AND variation (e.g. standard deviation) or associated estimates of uncertainty (e.g. confidence intervals)
☐	☒	For null hypothesis testing, the test statistic (e.g. *F*, *t*, *r*) with confidence intervals, effect sizes, degrees of freedom and *P* value noted<br>*Give P values as exact values whenever suitable.*
☒	☐	For Bayesian analysis, information on the choice of priors and Markov chain Monte Carlo settings
☒	☐	For hierarchical and complex designs, identification of the appropriate level for tests and full reporting of outcomes
☒	☐	Estimates of effect sizes (e.g. Cohen's *d*, Pearson's *r*), indicating how they were calculated

Our web collection on statistics for biologists contains articles on many of the points above.

Software and code

Policy information about availability of computer code

Data collection	Leica Application Suite X 3.4.2.18368, Leica MM AF 1.8.0, Image Reader LAS-4000 v2.0, STORM Scanner Control v5.0, Typhoon Scanner Control v5.0, Fast Real-Time PCR 7500 Software v2.3, Xia2 (Winter, 2010)
Data analysis	Fiji (Schindelin et al., 2012), MetaMorph 7.7.5.0, Oufti (Paintdakhi et al., 2016), MATLAB R2016b, Sigmaplot 12.5, Microsoft Excel 2013, MEGA 7.0.26 (Kumar et al., 2016), EMBOSS Needle (Li et al., 2015), NITPIC 1.2.274 (Scheuerman et al., 2015), SEDPHAT 12.1b75 (Brautigam et al., 2016), GUSSI 1.3.276 (Brautigam et al., 2015), PLGS (Waters), DynamX 3.0 (Waters), PHENIX (Adams et al., 2010), COOT (Emsley et al., 2010), PDB-REDO (Joosten et al., 2014), CLC workbench 12.0 (Qiagen), samtools (option 'mpileup', Version 1.9, Li, 2009).<br>The MATLAB scripts used in the asnalysis of images from fluorescence microscopy and ChIP-seq data are available from the corresponding author upon request.

For manuscripts utilizing custom algorithms or software that are central to the research but not yet described in published literature, software must be made available to editors/reviewers. We strongly encourage code deposition in a community repository (e.g. GitHub). See the Nature Research guidelines for submitting code & software for further information.

Data

Policy information about availability of data

All manuscripts must include a data availability statement. This statement should provide the following information, where applicable:

- Accession codes, unique identifiers, or web links for publicly available datasets
- A list of figures that have associated raw data
- A description of any restrictions on data availability

Co-ordinates and structure factors have been deposited with the RCSB: accession codes of 6SBW (form one) and 6SBX (form two). The ArrayExpress accession number for the ChIP-seq experiment is E-MTAB-8535. The PRIDE accession number for the HDX experiment is PXD018028. The source data underlying Figures 1b,d, 4a,c,d,e, 6a,c,d, 7a-e, 8a,c and Supplementary Figures 3b-d, 4a,d,e, 5, 6b, 7a, 8a,b are provided as a Source Data file.

Field-specific reporting

Please select the one below that is the best fit for your research. If you are not sure, read the appropriate sections before making your selection.

[x] Life sciences   [ ] Behavioural & social sciences   [ ] Ecological, evolutionary & environmental sciences

For a reference copy of the document with all sections, see nature.com/documents/nr-reporting-summary-flat.pdf

Life sciences study design

All studies must disclose on these points even when the disclosure is negative.

Sample size	No statistical methods were used to determine sample sizes. Sample size was based on our standard practice and increasing the sample size did not change the outcome of the experimets. Hypothesis testing indicated significant differences between conditions/strains and therefore no further data was acquired. Further data acquisition was not performed with the aim of achieving statistical significance for small differences in the results.
Data exclusions	No data were excluded.
Replication	All experiments were successfully replicated. The nature and number of replicates is indicated in the corresponding figure legend. Informations provided in Material and Methods section are sufficient to reproduce all the experiments. All single cell data presented is representative of the population.
Randomization	All single cell experiments were based on a random cells selection and thus no further data randomization was required.
Blinding	No blinding was performed because the acquisition and analysis required human intervention.

Reporting for specific materials, systems and methods

We require information from authors about some types of materials, experimental systems and methods used in many studies. Here, indicate whether each material, system or method listed is relevant to your study. If you are not sure if a list item applies to your research, read the appropriate section before selecting a response.

Materials & experimental systems

n/a	Involved in the study	
[ ]	[x]	Antibodies
[x]	[ ]	Eukaryotic cell lines
[x]	[ ]	Palaeontology
[x]	[ ]	Animals and other organisms
[x]	[ ]	Human research participants
[x]	[ ]	Clinical data

Methods

n/a	Involved in the study	
[ ]	[x]	ChIP-seq
[x]	[ ]	Flow cytometry
[x]	[ ]	MRI-based neuroimaging

Antibodies

Antibodies used	Antibodies used in this study include:<br>Rabbit polyclonal anti-FLAG antibody (Rockland, 600-401-383) (dilution 1:2000)<br>Rabbit polyclonal anti-mCherry antibody (Biovision, 5993-100) (dilution 1:10000)<br>Rabbit polyclonal anti-PilC antibody (dilution 1:5000) (Bulyha et al., 2009)<br>Secondary goat anti-rabbit IgG-peroxidase antibody (Sigma-Aldrich, A9044) (dilution 1:10000)
Validation	Data provided in the manuscript confirmed specificity of used antibodies, α-mCherry specificaly reacts with CdbA-mCherry, CdbA K8A/S10A-mCherry and CdbA R27A/R30A-mCherry (Figure 4d and 7d); α-FLAG with His6-CdbA-3xFLAG and CdbA-3xFLAG (Supplementary Figure 4e), α-PilC with PilC (validation provided by Bulyha et al., 2009)<br><br>Antibodies specificity was also confirmed by the manufacturer:<br>α-mCherry (https://www.biovision.com/mcherry-antibody.html)<br>α-FLAG (https://rockland-inc.com/store/Antibodies-to-FLAG-and-Antibodies-to-6XHIS-Tags-600-401-383-O4L_23854.aspx)

ChIP-seq

Data deposition

☒ Confirm that both raw and final processed data have been deposited in a public database such as GEO.

☒ Confirm that you have deposited or provided access to graph files (e.g. BED files) for the called peaks.

Data access links
May remain private before publication.

http://www.ebi.ac.uk/arrayexpress/experiments/E-MTAB-8535

Files in database submission

4043_WT1_run579_TACATCGG_S182_L002_R1.fastq.gz
4043_WT1_run579_TACATCGG_S182_L002_R2.fastq.gz
4043_WT2_run579_ATCGTCTC_S183_L002_R1.fastq.gz
4043_WT2_run579_ATCGTCTC_S183_L002_R2.fastq.gz
4043_CdbA1_run579_CCAACACT_S184_L002_R1.fastq.gz
4043_CdbA1_run579_CCAACACT_S184_L002_R2.fastq.gz
4043_CdbA2_run579_TCTAGGAG_S185_L002_R1.fastq.gz
4043_CdbA2_run579_TCTAGGAG_S185_L002_R2.fastq.gz
4043_ParB1_run579_CTCGAACA_S186_L002_R1.fastq.gz
4043_ParB1_run579_CTCGAACA_S186_L002_R2.fastq.gz
4043_ParB2_run579_ACGGACTT_S187_L002_R1.fastq.gz
4043_ParB2_run579_ACGGACTT_S187_L002_R2.fastq.gz
4043_input_WT_run579_CTAAGACC_S188_L002_R1.fastq.gz
4043_input_WT_run579_CTAAGACC_S188_L002_R2.fastq.gz
4043_input_CdbA_run579_AACCGAAC_S189_L002_R1.fastq.gz
4043_input_CdbA_run579_AACCGAAC_S189_L002_R2.fastq.gz
4043_input_ParB_run579_CCTTAGGT_S190_L002_R1.fastq.gz
4043_input_ParB_run579_CCTTAGGT_S190_L002_R2.fastq.gz
4043_WT1_run579_paired_trimmed_dedup.bam
4043_WT1_run579_paired_trimmed_dedup.bam
4043_WT2_run579_paired_trimmed_dedup.bam
4043_WT2_run579_paired_trimmed_dedup.bam
4043_CdbA1_run579_paired_trimmed_dedup.bam
4043_CdbA1_run579_paired_trimmed_dedup.bam
4043_CdbA2_run579_paired_trimmed_dedup.bam
4043_CdbA2_run579_paired_trimmed_dedup.bam
4043_ParB1_run579_paired_trimmed_dedup.bam
4043_ParB1_run579_paired_trimmed_dedup.bam
4043_ParB2_run579_paired_trimmed_dedup.bam
4043_ParB2_run579_paired_trimmed_dedup.bam
4043_input_WT_run579_paired_trimmed_dedup.bam
4043_input_WT_run579_paired_trimmed_dedup.bam
4043_input_CdbA_run579_paired_trimmed_dedup.bam
4043_input_CdbA_run579_paired_trimmed_dedup.bam
4043_input_ParB_run579_paired_trimmed_dedup.bam
4043_input_ParB_run579_paired_trimmed_dedup.bam
reads_WT1.txt
reads_WT1.txt
reads_WT2.txt
reads_WT2.txt
reads_CdbA1.txt
reads_CdbA1.txt
reads_CdbA2.txt
reads_CdbA2.txt
reads_ParB1.txt
reads_ParB1.txt
reads_ParB2.txt
reads_ParB2.txt
reads_input_WT.txt
reads_input_WT.txt
reads_input_CdbA.txt
reads_input_CdbA.txt
reads_input_ParB.txt
reads_input_ParB.txt
4043_WT1_annotated_peaks.bed
4043_WT1_annotated_peaks.bed
4043_WT2_annotated_peaks.bed

	4043_WT2_annotated_peaks.bed<br>4043_CdbA1_annotated_peaks.bed<br>4043_CdbA1_annotated_peaks.bed<br>4043_CdbA2_annotated_peaks.bed<br>4043_CdbA2_annotated_peaks.bed<br>4043_ParB1_annotated_peaks.bed<br>4043_ParB1_annotated_peaks.bed<br>4043_ParB2_annotated_peaks.bed<br>4043_ParB2_annotated_peaks.bed<br>enrichment_log2_WT1.txt<br>enrichment_log2_WT1.txt<br>enrichment_log2_WT2.txt<br>enrichment_log2_WT2.txt<br>enrichment_log2_CdbA1.txt<br>enrichment_log2_CdbA1.txt<br>enrichment_log2_CdbA2.txt<br>enrichment_log2_CdbA2.txt<br>enrichment_log2_ParB1.txt<br>enrichment_log2_ParB1.txt<br>enrichment_log2_ParB2.txt<br>enrichment_log2_ParB2.txt
Genome browser session<br>(e.g. UCSC)	No longer applicable

Methodology

Replicates	After chromatin shearing input aliquot was taken and each sample was split into two replicates for IP experiment.
Sequencing depth	2x150 bp, paired end read. On average about 3.5 million paired-end reads were collected for each sample.
Antibodies	Rabbit polyclonal anti-FLAG antibody (Rockland, 600-401-383)
Peak calling parameters	Peaks were identified using the CLC Genomics Workbench software (Qiagen) and sorted based on peak shape score. Peaks were considered significant if the enrichment in the IP sample over input was ≥4-fold.
Data quality	After sequencing, only high-quality paired-end reads were used for mapping. Only appropriately mapped paired-end reads were used for downstream analysis. Only peaks with >4 fold enrichment over input were analyzed.
Software	ChIP-seq signal enrichment across the genome was calculated with custom MATLAB scripts available on request.
